# Functional p53 is required for rapid restoration of daunorubicin-induced lesions of the spleen

**DOI:** 10.1186/1471-2407-13-341

**Published:** 2013-07-11

**Authors:** Lars Herfindal, Lene Myhren, Bjørn Tore Gjertsen, Stein Ove Døskeland, Gro Gausdal

**Affiliations:** 1 Department of Biomedicine, University of Bergen, Jonas Lies vei 91, 5009, Bergen, Norway; 2 Translational Signalling Group, Haukeland University Hospital, Jonas Lies vei 91, 5009, Bergen, Norway; 3 Institute of Medicine, Hematology Section, University of Bergen, PO Box 7804, 5020, Bergen, Norway

**Keywords:** Spleen, p53, p63, Daunorubicin, Lipofuscin, Apoptosis

## Abstract

**Background:**

The tumour suppressor and transcription factor p53 is a major determinant of the therapeutic response to anthracyclines. In healthy tissue, p53 is also considered pivotal for side effects of anthracycline treatment such as lesions in haematopoietic tissues like the spleen. We used a *Trp53*null mouse to explore the significance of p53 in anthracycline (daunorubicin) induced lesions in the spleen.

**Methods:**

Mice with wild type or deleted *Trp53* were treated with the daunorubicin (DNR) for three consecutive days. Spleens were collected at various time points after treatment, and examined for signs of chemotherapy-related lesions by microscopic analysis of haematoxylin-eosin or tunel-stained paraffin sections. Expression of death-inducing proteins was analysed by immunoblotting. Changes between *Trp53* wild type and null mice were compared by t-tests.

**Results:**

Signs of cell death (pyknotic nuclei and tunel-positive cells) in the white pulp of the spleen occurred earlier following DNR exposure in wt-mice compared to *Trp53*-null mice. While the spleen of wt-mice recovered to normal morphology, the spleen of the *Trp53*-null animals still had lesions with large necrotic areas and disorganised histologic appearance eight days after treatment. Immunoblotting showed that only *Trp53*-wt mice had significant increase in p21 after DNR treatment. However, both wt and null mice had elevated p63 levels following DNR exposure.

**Conclusions:**

p53 protects against severe and enduring cellular damage of the spleen parenchyma after DNR treatment, and initial DNR-induced apoptosis is not predictive of tissue lesions in the spleen. Our data indicate that p53 induction following DNR treatment serves to protect rather than to destroy normal tissue.

## Background

Numerous studies have demonstrated that the efficiency of DNA-damaging drugs in cancer therapy is dependent on the cellular status of the tumour suppressor factor *p53*[1-4]. The p53 pathway is often inactivated in human cancers, and deletions and mutations in *p53* are associated with progressive and more aggressive disease, and with poor prognosis and anthracycline resistance in several types of cancer [1,4-6]. In line with these results, there has been an increased focus on developing new drugs aiming to restore p53 activity in tumours [7-12]. However, the effect of p53 activation by drugs such as the anthracyclines on healthy tissue has to be considered in this respect, as induction of cell death and tissue damage in healthy tissue is an unwanted and severe side-effect of the anthracyclines.

It is known that anthracyclines cause lesions in haematopoietic tissues [13]. We therefore addressed the role of p53 in the toxic activity of the anthracycline daunorubicin (DNR) in the spleen, and compared the effect of DNR on the spleen in C57Bl/6 wild type (wt) and C57Bl6 *Trp53*-null mice. DNR induced more rapid cell death and loss of spleen weight in wild type (wt) compared to *Trp53*-null mice. However, whereas the *Trp53*-null mice had severe lesions of the spleen at day 4 after treatment, there was spleen structure recovery in *Trp53*-wt animals. Our data points to p53 as a protective factor in chemotherapy-induced normal tissue damage.

## Methods

### Mice

The *Trp53*-null mouse was generated by Jacks et al. [14], and was provided by Prof. Lozano, MD Anderson Cancer Center, Houston, TX, USA. *Trp53*-wt and null mice (C57BL/6) were generated by litter-mate inbreeding. Genotypes of weaned mice were determined by PCR analysis of DNA from an ear biopsy [14].

The mice used were male, and age matched. DNR (Sanofi-Aventis, Lysaker, Norway,) was administered intravenously (10 mg/kg) through the tail vein for three consecutive days. Control animals received relevant vehicle. Health status and weight of the mice were monitored daily. The mice experiments were approved by the Norwegian Animal Research Authority and conducted according to the European Convention for the Protection of Vertebrates Used for Scientific Purposes.

### Preparation and analysis of histological specimens

Spleens were excised from euthanized mice and washed in ice-cold PBS. Formalin-fixed tissues were embedded in paraffin, cut into 2-μm-thick sections and stained with haematoxylin and eosin (H&E). Terminal deoxynucleotidyl transferase-mediated dUTP-biotin nick end-labelling (TUNEL staining, In situ Cell Death Detection Kit, POD, Roche) was used for *in situ* staining of apoptotic DNA fragmentation. Pyknotic nuclei and cells containing lipofuscin-like pigments were assessed by microscopy of H&E-stained paraffin sections. The number of pyknotic nuclei in all the white pulp areas was counted and then divided by the number of white pulp regions.

The spleens were cut with scissors and cell suspensions were prepared by crushing the tissue pieces between two glass slides in PBS. Cell suspensions were filtered through a nylon cell strainer (40 μm), washed in PBS by centrifugation (160 × g, 6 min) and re-suspended at 0.5 × 10^6^ cells/ml in RPMI-1640 (Sigma-Aldrich Inc, St. Louis, MO) supplemented with 10% FCS (Gibco, Grant Island, NY). Cell death was assessed by flow cytometry after AlexaFluor 647-AnnexinV (Molecular Probes, Eugene, OR) and propidium iodide (PI) labelling. At least 30 000 non-gated live cell events were collected for each sample on an AccuriC6 cytometer (Ann Arbor, MI). Cells positive for AnnexinV alone or together with PI were counted as dead (apoptotic or necrotic). Untreated cells had less than 15% spontaneous cell death, and this was subtracted from the data on anthracycline-treated cells.

The data was compared by one-way analysis of variance (ANOVA) using IBM SPSS Statistics for Mac (version 19.0; IBM Corp.: Armonk, NY, 2010).

### Immunoblotting

Protein lysates were prepared from excised spleens, snap-frozen in liquid N_2_ and stored at -80°C. Tissue was grinded with a pestle and lysed in RIPA buffer supplemented with Complete mini protease inhibitor (Roche Diagnostics, Mannheim, Germany). The relative protein concentration was determined by Bradford and adjusted by Coomassie staining, and immunoblotting was as described [15]. Primary antibodies were from Santa Cruz Biotechology (Santa Cruz, CA, USA; p21, p63, Bax), and Imgenex (San Diego, CA, USA; p73) and secondary alkaline-phosphatase-conjugated antibody (a-3687 and a-3562) were from Sigma. CDP-Star substrate was from Tropix (Bedford, MA, USA). Chemiluminescence was detected using a Luminescent Image Analyser Apparatus (LAS 3000, FujiFilm, Tokyo, Japan) and Image Gauge Software (FujiFilm, Tokyo, Japan).

## Results and discussion

**Figure 1:**
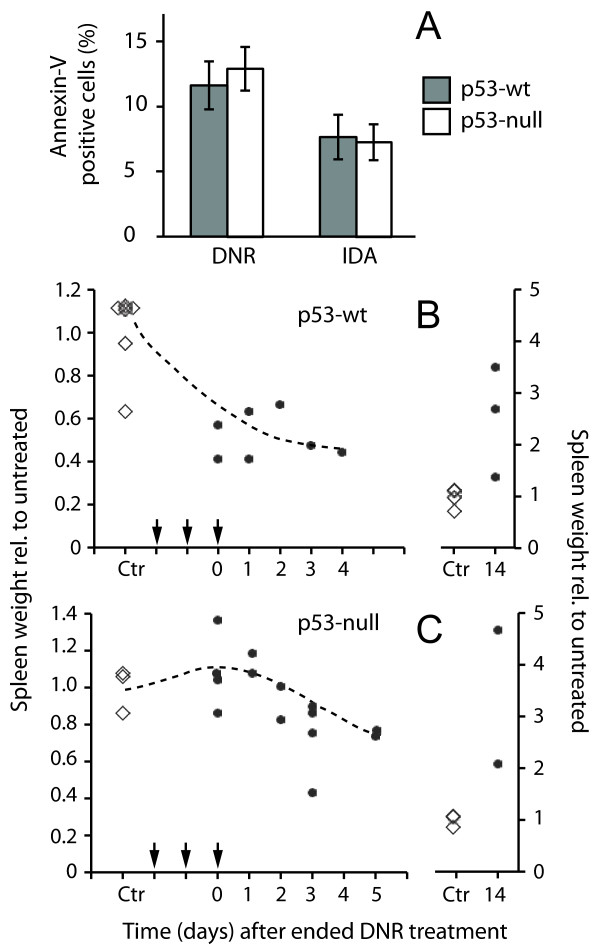
**DNR treatment reduces spleen weight in wt and p53-null mice. (A)**  Cells isolated from the spleen of *Trp53*-wt and -null mice were treated for 7 h with DNR (10 μM) or IDA (0.5 μM). Cells were stained with AnnexinV and PI, and analysed by flow cytometry. Data are given as mean and SEM, n = 3. **(B,C)***Trp53*-wt and -null mice were treated for 3 consecutive days with vehicle or 10 mg/kg DNR, and the spleen mass recorded and related to total animal weight at the given time points. The plots show relative spleen weight in treated animal to untreated animals. The arrowheads indicate days of DNR treatment. Diamonds represent untreated animals, and dots represent DNR-treated animals. The plots to the right show increase in spleen weight of DNR-treated animals 14 days after treatment. Note the difference in the scale between the left and right vertical axes. Each symbol represents one animal.

Since p53 status is often coupled to therapy response to anthracyclines like daunorubicin (DNR) and idarubicin (IDA) [5], we examined the effect of anthracyclines on splenocytes and spleen histology. We first studied if p53-status affected the *in vitro* response to the anthracyclines daunorubicin (DNR) and idarubicin (IDA) in cells isolated from the spleen, since p53 deficiency is often coupled to anthracycline resistance [5]. Both DNR and IDA are used as part of the standard treatment regime in leukaemia. We found that both drugs induced cell death to a similar degree insplenocytes from both wt and *Trp53*-null mice (Figure 1A). Hence, lack of p53 did not significantly seem to render the splenocytes resistant to anthracycline-induced death *in vitro*.

We next studied the *in vivo* effect of DNR treatment on the intact spleen in wt and *Trp53*-null mice. A typical therapy regime for AML patients consists of multiple 1–3 hour infusions of DNR during 3–6 days [16]. To study how p53 is involved in the drug-induced damage and recovery of the spleen, we administered DNR (10 mg/kg) i.v. to the mice every day for three days. Whereas spleen weight reduction was evident two days after onset of DNR-treatment in wt-mice (Figure 1B), in the *Trp53*-null mice reduced spleen weight was not observed until about five to six days after onset of treatment (Figure 1C). Also, weight reduction was more prominent in the wt mice compared to *Trp53*-null animals. A p53-dependent decrease in spleen mass has similarly been reported by others after ionising radiation [17]. Two weeks after treatment, there was an increase in spleen weight in both wt- and *Trp53*-null mice treated with DNR (Figure 1B,C; right plot).

**Figure 2:**
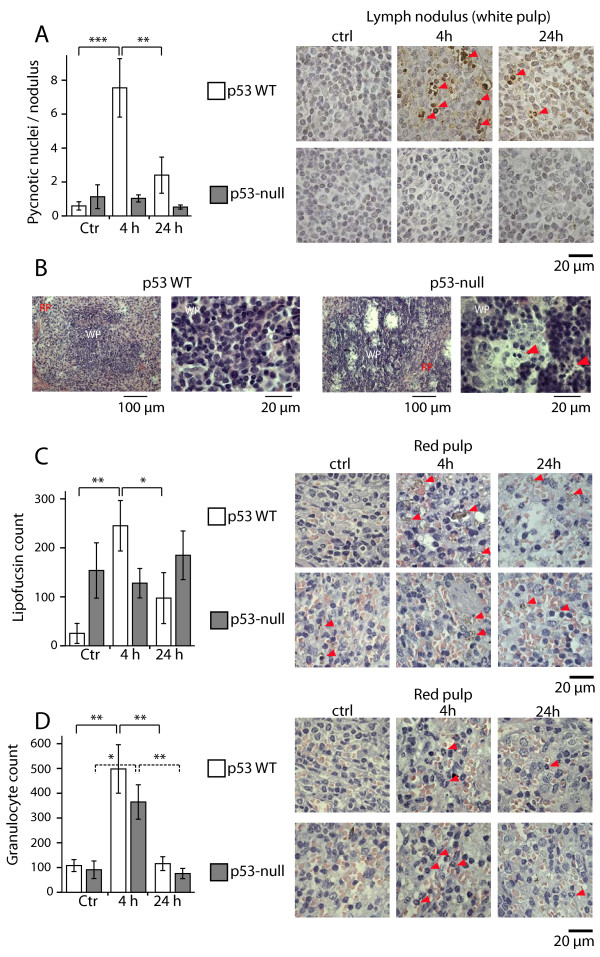
**DNR-induced apoptosis in the spleen is p53 dependent.**  Wt and *p53*-null mice were treated with vehicle (ctrl) or 10 mg/kg DNR for 3 days. 4 and 24 h after the last DNR injection, the spleens were removed, fixed and processed for paraffin sectioning for histological examination as described in the Experimental section. **(A)** Presence of pyknotic nuclei in the lymph nodules/white pulp in the spleen (bar diagram), or cell death visualised by TUNEL staining (right panels). The data represent the average number of pyknotic nuclei/lymph nodule. The data in the diagrams in **(A,C,D)**  are mean ± SEM, n = 3-7 mice. **(B)**  Haematoxylin- and eosin (H&E) stained paraffin sections of the spleen from *Trp53-*wt and -null mice four days after the last DNR injection. RP = red pulp, WT = white pulp, arrowheads indicate pyknotic nuclei. **(C)**  Presence of lipofuscin-like pigments in the red pulp of the spleen. The diagram shows the average number of cells with lipofuscin-like pigments per 400 μm^2^  of red pulp. H&E-stained sections from red pulp are shown in the panel to the right. The arrowheads indicate cells with lipofuscin-like pigments. **(D)**  Content of mature or maturing granulocytes, identified by nuclear morphology in the spleen. The diagram represents the average number of polymorphonuclear cells per 400 μm^2^  of red pulp. The right panels show typical appearance of granulocytes (arrowheads) in the red pulp of wt and p53-null mice. Asterisks indicate p < 0.05 (*), p < 0.01(**) or p <0.005 (***), one-way ANOVA.

We suspected that the early reduction in spleen weight could be due to cell death in the spleen. Accordingly, we found two hallmarks of cell death in spleens from wt-mice: 4 hours after the last treatment, the white pulps were scattered with i) pyknotic nuclei (Figure 2A, left panel), which corresponded with an increase of ii) TUNEL-positive nuclei (Figure 2A, right panels). The presence of both pyknotic and TUNEL-positive nuclei decreased during the next 20 hours (Figure 2A). Spleens from *Trp53*-null mice had no cells with pyknotic or TUNEL-positive nuclei in the white pulp 4 or 24 hours after DNR treatment (Figure 2A), suggesting that this early cell death was p53-dependent. Hence, a late onset of spleen weight reduction in *Trp53*-null mice corresponds to lack of early induction of cell death.

However, when we studied spleens from 3 *Trp53-*wt and 4 -null mice 4 days after the last DNR injection, we found pyknotic nuclei and gross pathological lesions in histological sections in both red and white pulp of the spleen only in the *Trp53*-null mice (Figure 2B). At this time, the wt mice had established normal spleen morphology with little or no signs of cell death (Figure 2B). Thus a late wave of p53-independent cell death seems to appear in the spleen of the *Trp53*-null mice. This later wave of cell death coincides with decreased spleen weight (Figure 1C).

We also found signs of DNR-induced cell death in the red pulp of the wt-mice. An increasing number of cells containing lipofuscin-like pigments were detected 4 hours after the last DNR injection (Figure 2C). Elevated levels of lipofuscin-like pigments have been found in the spleen of mice subjected to ionising radiation [18], and could be due to accumulation of non-degradable debris in for instance macrophages [19]. The number of cells positive for lipofuscin-like pigments decreased during the next 20 hours (Figure 2C), as was seen for pyknosis and TUNEL-positive cells (Figure 2A). Interestingly, *Trp53*-null mice had high numbers of cells containing lipofuscin-like pigments both in the red pulp (Figure 2C) and in the white pulps (not shown), and treatment with DNR did not increase the number of cells containing lipofuscin-like pigments (Figure 2C). This suggests that natural turnover of cells in the spleen of *Trp53*-null mice leave degradation products such as lipofuscin-like pigments.

Four hours after completed DNR treatment we also detected a 4–5 fold increase in the number of mature and maturing polymorphonuclear cells in the red pulp both in *Trp53*-wt and null mice (Figure 2D). Stem cells and progenitors have been reported to migrate between bone marrow and spleen after induction of haematopoietic cell stress [20]. This migration could be a response to bone marrow deprivation after DNR treatment, and indicate that the spleen red pulp partly replaces haematopoietic functions after extensive DNR treatment.

**Figure 3:**
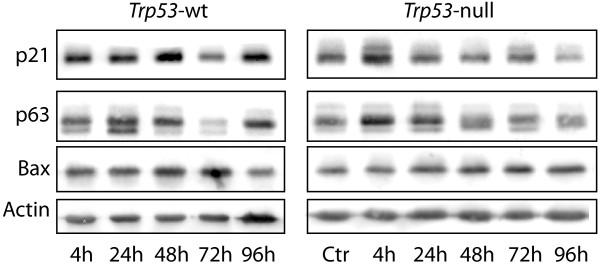
**Expression of p21, p63 and Bax in the spleen after DNR treatment.**  Protein extracts from spleens excised from animals before or after treatment with DNR at the indicated time-points were analysed for levels of p21, p63 or Bax by immunoblotting, as described in the methods section. Actin was used as loading control.

The late wave of cell death that we observed in the *Trp53*-null mice (Figure 2B) has similarly been reported to occur in the intestine of the *Trp53*-null mice after gamma-irradiation and has been assigned to induction of mitotic catastrophe due to lack of p53-induced cell cycle arrest [21]. We therefore analysed spleens from DNR-treated *Trp53-*wt and -null mice for p21 induction. Immunoblotting showed elevated expression of p21 in spleens from wt-mice at 24 and 48 hours after DNR treatment (Figure 3, left panel) similar to what is seen with other DNA-damaging agents [22,23]. The *Trp53* null mice had only modest increase in p21 levels (Figure 3, right panel). The early elevation in p21 in the spleen from wt-mice could offer protection against severe tissue damage by induction of transient cell cycle arrest that allows the cells to repair drug-induced DNA damage and hence protect against mitotic catastrophe.

p63 is, together with p73, shown to be crucial for p53-meidated cell death after DNA damage [24], and can increase Bax expression and sensitise cells to apoptotic stimuli [25]. We found that p63 and to some degree Bax was elevated in spleens from wt-mice at 24 and 48 hours after DNR treatment (Figure 3), the same time points where there was an increase in apoptotic nuclei and lipofuscin-like pigments (Figure 2A and C). We did not find any change in the expression of p73 neither in *Trp53*-wt nor null mice (data not shown). The *Trp53*-null mice had a prolonged increase of p63 and Bax, which lasted until 96 hours after termination of DNR treatment (Figure 3). This coincides with the late wave of p53-independent cell death that appeared in the spleen of the *Trp53*-null mice. It thus appears that in addition to lack of early p21-mediated cell cycle arrest (eventually resulting in mitotic catastrophe), the late massive cell death seen in the spleen of *Trp53*-null mice (Figure 2B, right panel), but not in *Trp53*-wt mice (Figure 2B, left panel) could also be mediated by up-regulation of p63 and Bax in the absence of p53.

## Conclusion

This report indicates an anthracycline-induced early p53-dependent cell death in the spleen. In the *Trp53*-wt mice, the spleen appeared to recover after DNR treatment with no histopathological signs of cell death or tissue deterioration present four days after end of treatment. However, *Trp53*-null mice suffered from large lesions in the spleen parenchyma corresponding to a later induction of p53-independent cell death. These findings have clinical implications for therapy aiming to restore p53-dependent cell death pathways in cancer cells with non-functional p53. The efficacy of this therapy approach is debated [26], and the response apparently varies between drugs [27]. We show here that restoration of p53 activity does not damage the anthracycline-sensitive spleen, but may rather serve to protect this during intensive chemotherapy.

## Competing interests

The authors declare that there are no competing interests.

## Authors’ contributions

LH and GG: Designed the study and executed mouse experiments, cell death study and histopathological analyses. Prepared manuscript draft. LM: Designed and executed WB analyses, drafted manuscript. SOD and BTG: Study design, data interpretation and drafted manuscript. All authors read and approved the final manuscript.

## Pre-publication history

The pre-publication history for this paper can be accessed here:

http://www.biomedcentral.com/1471-2407/13/341/prepub
